# Orientation dependence and decay characteristics of T_2_* relaxation in the human meniscus studied with 7 Tesla MR microscopy and compared to histology

**DOI:** 10.1002/mrm.27443

**Published:** 2018-09-30

**Authors:** Benedikt Hager, Sonja M. Walzer, Xeni Deligianni, Oliver Bieri, Andreas Berg, Markus M. Schreiner, Martin Zalaudek, Reinhard Windhager, Siegfried Trattnig, Vladimir Juras

**Affiliations:** ^1^ Department of Biomedical Imaging and Image‐guided Therapy High Field MR Centre, Medical University of Vienna Vienna Austria; ^2^ CD Laboratory for Clinical Molecular MR Imaging Vienna Austria; ^3^ Austrian Cluster for Tissue Regeneration Ludwig Boltzmann Institute for Experimental and Clinical Traumatology Vienna Austria; ^4^ Department of Orthopedics and Trauma Surgery Medical University of Vienna Vienna Austria; ^5^ Division of Radiological Physics, Department of Radiology University of Basel Hospital Basel Switzerland; ^6^ Department of Biomedical Engineering, University of Basel Allschwil Switzerland; ^7^ Center for Medical Physics and Biomedical Engineering Medical University of Vienna Vienna Austria; ^8^ Department of Imaging Methods, Institute of Measurement Science Slovak Academy of Sciences Bratislava Slovakia

**Keywords:** biexponential, histology, magic angle, meniscus, T2* mapping, variable echo time

## Abstract

**Purpose:**

To evaluate: (1) the feasibility of MR microscopy T_2_* mapping by performing a zonal analysis of spatially matched T_2_* maps and histological images using microscopic in‐plane pixel resolution; (2) the orientational dependence of T_2_* relaxation of the meniscus; and (3) the T_2_* decay characteristics of the meniscus by statistically evaluating the quality of mono‐ and biexponential model.

**Methods:**

Ultrahigh resolution T_2_* mapping was performed with ultrashort echo time using a 7 Tesla MR microscopy system. Measurement of one meniscus was performed at three orientations to the main magnetic field (0, 55, and 90°). Histological assessment was performed with picrosirius red staining and polarized light microscopy. Quality of mono‐ and biexponential model fitting was tested using Akaike Information Criteria and F‐test.

**Results:**

(1) The outer laminar layer, connective tissue fibers from the joint capsule, and the highly organized tendon‐like structures were identified using ultra‐highly resolved MRI. (2) Highly organized structures of the meniscus showed considerable changes in T_2_* values with orientation. (3) No significant biexponential decay was found on a voxel‐by‐voxel–based evaluation. On a region‐of‐interest–averaged basis, significant biexponential decay was found for the tendon‐like region in a fiber‐to‐field angle of 0°.

**Conclusion:**

The MR microscopy approach used in this study allows the identification of meniscus substructures and to quantify T_2_* with a voxel resolution approximately 100 times higher than previously reported. T_2_* decay showed a strong fiber‐to‐field angle dependence reflecting the anisotropic properties of the meniscal collagen fibers. No clear biexponential decay behavior was found for the meniscus substructures.

## 1 INTRODUCTION

Optimal meniscus function and integrity is of critical importance for the knee joint. Degeneration, tear and extrusion, and partial or full meniscectomy may lead to cartilage volume loss and put at risk for the subsequent development of premature knee osteoarthritis.^1^, ^2^ Therefore, noninvasive detection of early changes in the meniscal structure would be the prerequisite for identification of patients at risk for tear and further therapeutic decision making, to preserve meniscus tissue and delay early onset of the degenerative process of the knee joint. For cartilage, MRI increasingly caters to this need. However, the human meniscus—being of fibrocartilage tissue—is known to contain primarily short T_2_/T_2_* components. Hence, with conventional clinical MR sequences, the MR signal of healthy meniscal tissue decays too rapidly and appears hypointense or dark.^3^, ^4^ As a result, conventional MRI techniques, such as proton‐density–, T_2_– and T_1_–weighted imaging can only detect tears and late‐stage degenerations of the meniscus.

In recent years, advanced MRI imaging techniques, such as T_2_* mapping with ultrashort echo times or variable echo times (vTE), gained increasing interest,^5^, ^6^, ^7^ because they overcome limitations of conventional clinical sequences. In this context, it is of particular interest to establish correlations between quantitative T_2_* measurements and biological changes in the collagen alignment as well as in the extracellular matrix (ECM), both visualized with histochemical methods.

The rationale for investigating T_2_* values in highly ordered collagen fiber tissue relates to the relationship between T_2_* and the condition of the ECM; that is, the complex array of collagen, glycoproteins, and proteoglycans. Additionally, myxoid changes, fibrocartilaginous separation of the matrix, extensive fraying, and tears^8^ are accompanied by a loosening of collagen fiber organization and increasing water content^9^ and are found to increase T_2_* values.^6^, ^7^

Recent studies have suggested that T_2_* decay in the meniscal tissue can be described by a biexponential function, where the short and long component of T_2_* are suggested to reflect bound and free (bulk) water pools.^7^, ^10^ Moreover, it was found that the short component of T_2_* might contain additional information about the collagen matrix, and that it provides greater ability for distinguishing between normal and degenerated meniscus.^7^ Accordingly, high‐resolution mono‐ and biexponential quantitative T_2_* mapping is a method with great potential for noninvasive detection of structural and degenerative changes in meniscal tissue.^6^, ^7^ However, to date, the mechanisms of mono‐ and biexponential decay in human meniscus are still not fully understood.

The orientational collagen fiber to magnetic field angle dependence as a result of residual dipolar coupling is a well‐known property of highly ordered collagen structures, such as tendon,^11^, ^12^, ^13^, ^14^ cartilage,^15^ and meniscus.^16^ Dipolar interaction of protons in the collagen network is modulated by the term^17^ (Equation (1)):

(1) $$D∼3\cos^{2}\theta -1,$$

where θ is the collagen fiber to magnetic field angle (or short, fiber‐to‐field angle). At $\theta =55^{\circ },125^{\circ }$, etc., the so‐called magic angles, all water protons tend to resonate at the same frequency, which results in an increase of effective T_2_/T_2_* values.^17^ Orientation dependence of meniscus T_2_* values has not yet been evaluated.

The aims of this study were to: (1) show the feasibility of in vitro mono‐ and biexponential T_2_* analysis of degenerated human meniscus specimens using a 3D vTE sequence with microscopic pixel resolution and compare T_2_* results to histological findings; (2) use quantitative MR microscopy to investigate how T_2_* values within different meniscal zones vary depending on the circumferential fibers (the predominant fiber type making up the bulk of the meniscus, as previously described^18^, ^19^) to the main magnetic field; and (3) increase understanding of T_2_* decay characteristics and water compartmentalization in human meniscus by statistically evaluating the preference of a mono‐ versus a biexponential model on a voxel‐by‐voxel– as well as on a region‐of‐interest (ROI)–averaged basis.

## 2 METHODS

### 2.1 Sample preparation and MR methods

Meniscus specimens were obtained with written informed consent from two osteoarthritis (OA) patients with no documented meniscal lesions undergoing knee replacement surgery in accord with the terms of the ethics committee of the Medical University of Vienna (EK‐Nr: 1065/2011), following the guidelines of the Declaration of Helsinki and Tokyo.

All MRI experiments were performed on an ultra‐high‐field 7 Tesla (T) whole‐body system (Magnetom Siemens Healthineers, Erlangen, Germany), using a microimaging system^21^ providing a maximum gradient strength of 750 mT/m.

### 2.2 Experiment 1: correlation of T_2_* maps with histological assessment

In total, five meniscal segments were obtained from a pair of human lateral and medial menisci from the same OA knee joint (age: 60 years, female; Kellgren‐Lawrence Score: 4; Knee Society Score: 58/60), from which three representative segments were obtained from the medial meniscus (body, posterior horn, and anterior horn, further denoted as segments 1, 2, and 3, respectively) and two representative segments were obtained from body and the posterior horn of the lateral meniscus fragment (further denoted as segments 4 and 5, respectively).

For T_2_* mapping a 19‐mm ^1^H‐NMR volume coil (Rapid Biomedical, Wuerzburg, Germany) was used. For quantitative monoexponential T_2_* assessment, a 3D vTE sequence was used.^22^ The sequence is based on a gradient echo spoiled sequence, but was modified to use a highly asymmetric readout and a variable echo time approach in phase and slice encoding direction to dynamically adapt and shorten the echo time toward the center of k‐space. A short (150‐μs) nonselective hard radiofrequency (RF) pulse was applied for excitation and RF (phase cycling) and gradient spoiling were used to disrupt residual transverse magnetization and minimize repetition time. This concept allows fast high‐resolution measurements with effective echo times in the submillisecond range. In addition, the sequence uses projection onto convex sets formalism for reconstruction of the undersampled data set.^22^, ^23^

In this experiment, data for T_2_* maps were acquired using a series of single‐echo vTE scans. This was done to ensure that the T_2_* decay of the meniscus, especially the short echo range (around 0.5‐2.5 ms), was adequately covered by all echoes. Moreover, given the short T_2_* values of meniscus, these short echo times are critical to gain high signal‐to‐noise ratio (SNR).

To resolve the structures of the meniscus, the resolution along the triangular cross‐section of the meniscus was given priority. To this end, microscopic in‐plane pixel resolution (<100 μm) with high SNR, that is needed for T_2_* mapping, was achieved at the expense of lower slice resolution (slice thickness = 400 µm) along the circumferential collagen fiber direction (i.e., the third dimension of the voxel).

The following echo times (TEs) were used: 0.4, 0.7, 1.2, 1.7, 2.5, 4, 6, 8, and 12 ms. Other imaging parameters were: field of view (FOV) = 12 × 14 mm^2^, pixel size = 60 × 60 μm^2^, slice thickness = 0.4 mm, number of slices = 22, repetition time (TR) = 25 ms, flip angle (FA)^24^ = 9°, signal averages = 4, and acquisition duration = 90 minutes.

### 2.3 Experiment 2: fiber‐to‐field angle dependence

Anisotropic analysis was performed on one human lateral meniscus fragment (age: 64 years, female; Kellgren‐Lawrence Score: 4; Knee Society Score: 42/50, posterior horn was removed during surgery), using the same 7 T microimaging setup. In contrast to the previous measurement, a 39‐mm ^1^H‐NMR volume coil (Rapid Biomedical) was used. In order to image the meniscus specimen in different orientations, it was fastened on a plastic cross using thread and then imbedded in the middle of a 30‐mm‐diameter plastic sphere filled with physiological saline solution. For morphological evaluation of the meniscus structure, a proton‐density‐weighted spin‐echo sequence was used. Imaging parameters were: TE = 6.4 ms, TR = 3500 ms, FA = 180°, FOV = 30 × 30 mm^2^, matrix = 448 × 448, pixel size = 67 × 67 μm^2^, slice thickness = 0.4 mm, slice offset = 100%, and number of slices = 14.

Mono‐ and biexponential T_2_* assessment was performed on data acquired with the 3D vTE sequence. In this experiment, a multi‐echo approach was applied. Image parameters included: 12 TEs = 0.82, 1.82, 2.82, 7.23, 9.23, 11.23, 13.39, 15.39, 17.39, 19.55, 21.55, and 23.55 ms, TR = 38 ms, FA = 17°, FOV = 30 × 30 mm^2^, matrix = 448 × 448, pixel size = 67 × 67 μm^2^, slice thickness = 0.4 mm, number of slices = 72, signal averages = 1, and acquisition duration = 48 minutes. Water selective binomial 1‐1 excitation (a pair of 150‐µs pulses) was used to null possible signal from the fat.^25^, ^26^ All measurements (PD‐weighted, T_2_* mapping) were performed in both axial and coronal direction of the meniscus. The sample was positioned in a way to be reasonably sure that the orientation of most of the circumferential fibers with respect to the main magnetic field was: 0°, 55°, and 90°.

These angles were chosen for the following reasons: At 0°, the dipolar interaction is at a maximum and all water protons related to collagen fibers tend to resonate at different frequencies, which results in expected lowest effective T_2_ and T_2_* values. In contrast, at the magic angle (55°), all water protons tend to resonate at the same frequency, which results in the expected highest effective T_2_/T_2_* values.^17^ The 90° angle was chosen because it reflects a similar angle to the in vivo case of the meniscus in a horizontal MR scanner.

In this regard, the plastic sphere and the cross, where the meniscus fragment is fastened on, were used for positioning and alignment of the FOV and thus the slices, ensuring equally aligned slices for each orientation.

ROIs were defined in 3 different areas (tendon‐like tissue, fibrous tissue from the external circumference, and fibrous tissue from the internal circumference) representing the variability in human meniscus. Subsequent ROI analysis (voxel‐wise– and on an ROI–averaged basis) was performed on 10 consecutive slices from the meniscus body in 3 orientations and for all sets of ROIs.

### 2.4 Image postprocessing

Calculation of mono‐ and biexponential T_2_* analysis was performed using a nonlinear Levenberg‐Marquardt (LM) algorithm curve‐fitting method,^27^, ^28^ which was conducted using a custom‐built IDL script (Interactive Data Language; Research Systems, Inc, Boulder, CO), using the mpcurvefit library,^29^ and was done on a voxel‐by‐voxel basis with a confidence interval of 95%.

For monoexponential fitting, a 3‐parameter model fitting was used (Equation (2))):

(2) $$S_{m}=A_{1}e^{\frac{-TE}{T_{2}^{*}}}+\varepsilon ,$$

where *A*_1_ is the maximum signal at t = 0, which is also often described as a product of proton density (S_0_) and a proportionality constant (k) subsuming signal gain or attenuation by the scanner's hard‐/software* (A*_1 _=_ _k S_0_). T_2_* corresponds to the actual monoexponentially calculated* T*_2_* value, and ε is the offset. The offset can be seen as a non‐zero baseline that takes into account the signal that has not converged toward zero.^30^

The initial parameter value for the monoexponential T_2_* component was given as T_2_* = 10 ms, which should be a good estimation according to literature.^7^ It should be noted, however, that the LM fit is reasonably insensitive to the starting values of the parameters.^31^ Therefore, if initial values are slightly off, usually the fit still leads to good results, which is an important property for this evaluation, because the meniscus is a very heterogeneous structure with different T_2_* values.

The same MRI data were also fitted by a biexponential decay curve, using a 5‐parameter model function (Equation (3)):

(3) $$S_{m}=B_{1}e^{\frac{-TE}{T_{2\;s}^{*}}}+B_{2}e^{\frac{-TE}{T_{2\;l}^{*}}}+\varepsilon$$

where *T*_2_*_s_ refers to the short *T*_2_* component and *T*_2_*_I_ to the long component of *T*_2_*. B*_1_* and B*_2_* are the component ratios. ε is the baseline offset given primarily by noise.

Initial parameter values for the biexponential T_2_* components were set to *T*_2_*_s_ = 2 ms for the short T_2_* component and *T*_2_*_I_ = 15 ms for the long component based on a priori information from the literature.^7^, ^10^ Moreover, lower and upper boundary constraints of the parameters were chosen, where for short components the limits are: [0, 20 ms], and for the long components: [0, 200 ms].

In order to test whether individual meniscus voxels show mono‐ or biexponential T_2_* decay, we compared the quality of mono‐ and biexponential model fitting using Akaike information criteria (AIC).^32^

In general, adding supplementary parameters allows to increase the likelihood of a model, but this introduces the possibility of overfitting.^33^ In other words, the model with fewer parameters will almost always fit the data worse. Consequently, AIC adds a penalty term for the number of parameters. In our specific case, the number of data points (n = 12) is rather low compared to the number of parameters (k = 3 and 5, for mono‐ and biexponential model, respectively); therefore, a small‐sample (second order bias corrected) Akaike information criterion (AIC_C_) was used.^34^ Assuming that the scatter of data points around the best fitted curve follows a Gaussian or normal distribution with constant variance (which is usually assumed in nonlinear regression), then the AIC_C _can be given as follows (Equation (4)):

(4) $${\mathrm{AIC}}_{C}=2k+n\text{log}\left(\frac{\mathrm{SSE}}{n}\right)+\frac{2k(k+1)}{n-k-1},$$

where n refers to the number of data points, k is the number of parameters, and SSE is the standard error of the estimate. The model with the lower AIC_C_ is more likely the one being correct.

Additionally, we also used F‐tests to compare the goodness of fit of the mono‐ and biexponential model and assess, for each voxel, whether a biexponential model is more appropriate over a monoexponential model. This was evaluated by comparing the standard error of the estimate adjusted for the number of degrees of freedom^35^ (Equation (5)):

(5) $$F=\frac{\left({\mathrm{SSE}}_{\mathrm{mono}}-{\mathrm{SSE}}_{\mathrm{bi}}\right)}{{\mathrm{SSE}}_{\mathrm{bi}}}\frac{v_{\mathrm{bi}}}{v_{\mathrm{mono}}-v_{\mathrm{bi}}}$$

where SSE_mono_ and SSE_bi _are the standard error of the estimate (SSE) of the mono‐ and biexponential fits, respectively, and v_mono_ and v_bi _are the degrees of freedom of both analyses (v = n – k). The *P* value of the associated F‐ratio was then calculated based on F‐distribution. All statistical analyses were performed setting the critical significance level to 5%. For a *P* value smaller than an α‐level of 0.05, the biexponential model was considered preferable. Vice versa, the monoexponential model was considered preferable. In contrast to AIC_C_, F‐test is a null‐hypothesis test that has the prerequisite that the models must be nested, which is the case for the mono‐ and biexponential models.

For each slice, F‐test and AIC_C_ were performed between a mono‐ and a biexponential model, and from the results the following maps are calculated: *T*_2_*_s_ map of the biexponential T_2_* analysis for the short relaxing component, *T*_2_*_I_ map of biexponential T_2_* analysis for the long component, a binary map depicting voxels that can be preferentially considered biexponential (0 = monoexponential decay, 1 = biexponential decay), and short and long T_2_* component fraction maps. Additionally, a monoexponential T_2_* map (T_2_*_m_) was calculated for each slice.

Moreover, F‐test and AIC_C_ were used to test whether individual selected regions of interest showed mono‐ or biexponential T_2_* decay type on an ROI–averaged basis.

### 2.5 Statistical analysis

Grand mean T_2_* (T_2_*_m_) values of all of the slices of the meniscus segments were calculated using the weighted mean values (weighted by number of voxels per slice) of the mean T_2_* of each slice.

From the ROIs of the 10 slices of the orientational analysis, mono‐ and biexponential T_2_* analyses were performed. Bartlett's test^36^ was used to test for heteroscedasticity. For a *P* value > 0.05, the variances are homogeneous and the data set is considered homoscedastic. Depending on the result of Bartlett's test, three‐way analysis of variance (ANOVA) and Welch's ANOVA were then performed to test for statistical significance. A *P* value ≤ 0.05 was considered to indicate statistically significant results. All statistical analysis was performed using R Statistical Software (version 3.2.3^37^; R Foundation for Statistical Computing, Vienna, Austria). Box plots were created using the *ggplot2* package^38^ and an extension, *ggsignif*.^39^

### 2.6 Histological assessment

Following the MR measurement, the triangle‐shaped meniscus cross‐sections were fixed with neutral‐buffered 4% formaldehyde and embedded in paraffin after decalcification. After deparaffination through xylene and graded alcohol, 2.5‐µm serial slices were stained with hematoxylin‐eosin, used for the morphological overview. Picrosirius red (PSR) staining was used for detection of collagen under light microscopy and visualizing collagen alignment by polarized light microscopy (PLM; Zeiss, Oberkochen, Germany). Under polarized light, birefringence of collagen fibers allows to define the collagen architecture of tissue. The combination of PSR and Alcian blue staining provides further information about local changes of increasing cartilaginous differentiation in the ECM during the process of degeneration.

## 3 RESULTS

**Figure 1:**
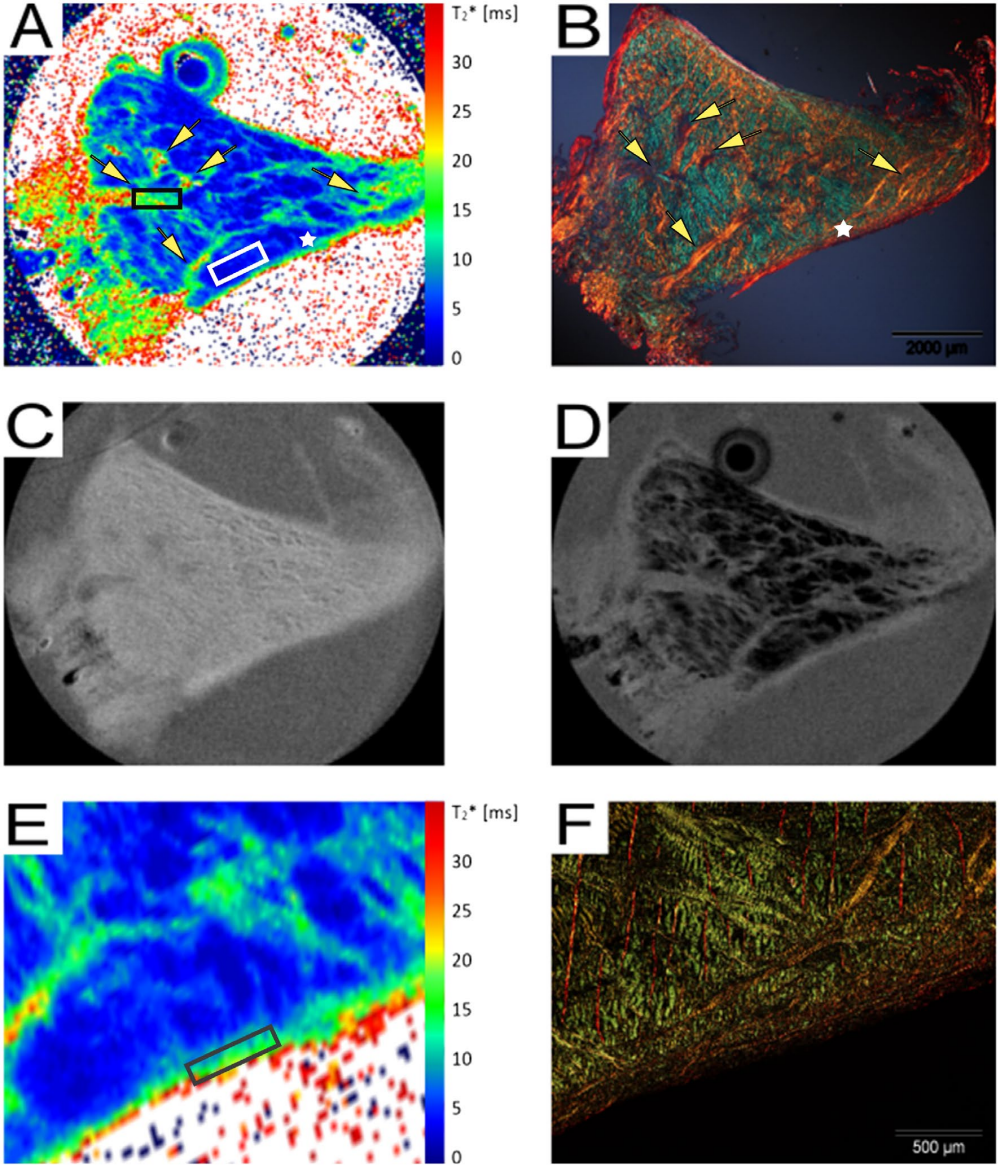
A, T_2_* map of the body of a degenerated medial human meniscus measured with a 3D vTE sequence. ROIs are depicted in the region of highly ordered circumferential fibers (ROI1, white) and fibrous tissue from the external circumference (ROI2, black). Yellow arrows show fibrous tissue. The white asterisk depicts the area that is shown as a close‐up in (E). B, Shows the corresponding PSR‐stained slice measured using polarized light filter with 10 × magnification. Again, yellow arrows show fibrous tissue and the white asterisk depicts the area that is shown as a close up in (F). C, The first echo image (TE = 0.4 ms) shows high signal intensity for all meniscal substructures. D, The eighth echo image (TE = 8 ms) depicts the fibrous network of the meniscus. The signal from the highly ordered circumferential fibers is almost completely gone with this echo time. E, Close‐up image of the T_2_* map depicts the lamellar‐like layer of the meniscus. F, Close‐up image of the PSR‐stained slice measured using polarized light (40 × magnification) indicates the outer lamellar‐like layer of the meniscus

Figure 1 shows a representative microscopic T_2_* map (Figure 1A) of one segment of the body of a medial meniscus (segment 1) and the corresponding PSR‐stained slice using a polarized light filter (Figure 1B). The first (TE = 0.4 ms) and the eighth echo image (TE = 8 ms) of the single‐echo sequence are shown in Figure 1C and 1D, respectively. This meniscus segment was measured with the circumferential fibers oriented at approximately 0° to the main magnetic field (B_0_). Noteworthy, there is a striking similarity between the T_2_* map, the eighth echo image, and the histological assessment of PSR staining under polarized light.

The first echo time image measured with TE = 0.4 ms (Figure 1C) shows high SNR (~35) for all of the meniscal microstructures, whereas, for instance, for an echo time of TE = 8 ms (Figure 1D), many parts of the meniscus (particularly highly ordered circumferential fibers) display little to no signal and provide a hypointense background for the fibrous network, which is well depicted with this echo time.

Qualitatively, the results show that thick fibrous bundles penetrate through the meniscus cross‐section from the outer vascularized zone into the inner avascular zone. As mentioned earlier, this meniscus sample was measured with the circumferential fibers parallel to the main magnetic field. With this fiber‐to‐field orientation and setup, the overall mean T_2_* value of segment 1 is 7.9 ± 4.5 ms.

In this sample, circumferential fiber bundles show relatively short T_2_* values as depicted by an ROI assessment (ROI1, white rectangle in Figure 1A), which shows a mean T_2_* value of 4.4 ± 0.9 ms. This region in the external circumference is further denoted as a tendon‐like region, because it histologically resembles to tendon tissue.^19^

For the fibrous bundles, T_2_* values are much higher as exemplarily depicted by ROI2 (black rectangle) in Figure 1A, which shows a mean T_2_* value of 15.5 ± 3.9 ms. Other fibrous regions are depicted by yellow arrows (Figure 1A,B).

Moreover, the laminar outer layer, which has a thickness of around 200 µm,^19^ can clearly be visualized by this measurement setup as depicted by close‐up images of the T_2_* map and the PSR‐stained image measured with polarized light (Figure 1E and F, respectively). The position of the close‐up images is demarked by white asterisks in Figure 1A and B.

The T_2_* values of the laminar outer layer are higher compared to circumferential fibers as exemplarily depicted by an ROI (black rectangle) in the close‐up image of the T_2_* map (Figure 1E), which shows a mean T_2_* value of 11.7 ± 2.1 ms.

For all meniscus samples and slices from experiment 1, the percentage of voxels that can preferentially be considered biexponential is extremely low (<3%), when tested with AIC_C_ and F‐test. The results of experiment 1 are summarized in Table 1.

**Table 1:** Summary of mean T_2_* (T_2_*_m_) values and of percentages of voxels that can preferentially be considered to show biexponential decay

Segment nr. (voxel‐wise)	T_2_*_m_ [ms]	AIC_C_ [%]	F‐test [%]
SEG. 1	7.9 ± 4.5	<1%	<1%
SEG. 2	11.4 ± 7.7	2%	2%
SEG. 3	8.5 ± 5.3	<1%	<1%
SEG. 4	12.9 ± 4.9	<1%	<1%
SEG. 5	11.5 ± 6.3	<1%	<1%

**Figure 2:**
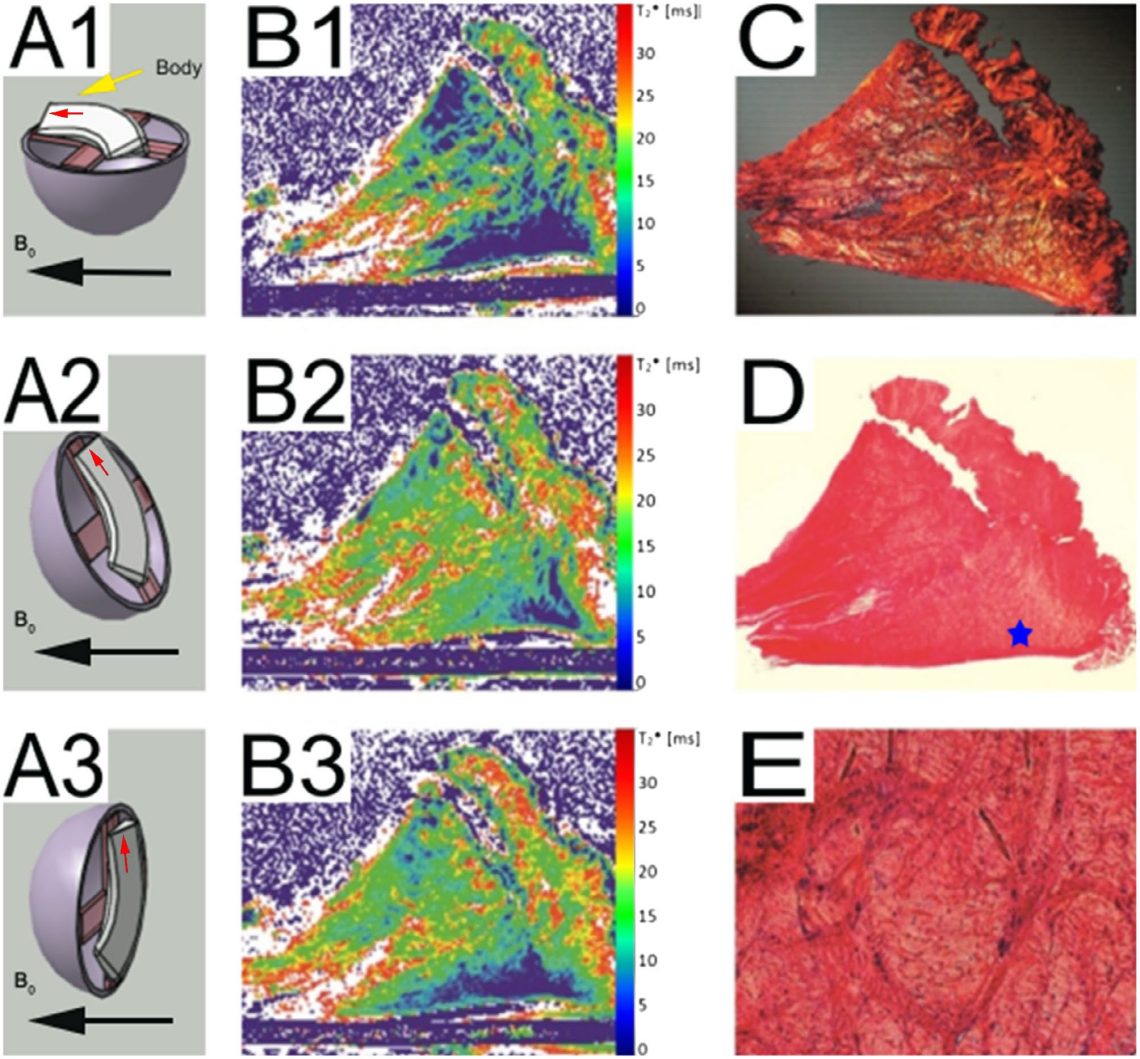
A1–A3, Schematic drawings of the fiber‐to‐field orientations: 0°, 55°, and 90° (A1, A2, and A3, respectively). The yellow arrow shows the position of the meniscus body, and the red arrows indicate the orientation of the circumferential fibers in this area. B1–B3, T_2_* maps of 1 representative zone from the body of the meniscus with fiber‐to‐field angle 0° (B1), 55° (B2), and 90° (B3). C, The corresponding PSR‐stained slice with polarized light visualizing the collagen fiber distribution. D, PSR/alzian blue combination stain was used for the visualization of collagen fibers and local increased appearance of glycosaminoglycans in fibrocartilaginous tissue. E, Shows a close‐up of the tendon‐like zone of the meniscus. The blue asterisk in D, depicts the area that is shown as a close‐up

Figure 2 depicts one meniscus specimen measured in three different orientations to the magnetic field. The schematic drawings of Figure 2 (A1, A2, and A3) show how the meniscus sample was measured with respect to the main magnetic field: 0° (Figure 2A1), 55° (Figure 2A2), and 90° (Figure 2A3). Figure 2 (B1, B2, and B3) shows T_2_* maps from the same representative zone from the body of the meniscus measured in the respective fiber‐to‐field angles.

The magic angle orientation (55°) showed highest T_2_* values with a grand mean value of 27.1 ± 1.0 ms, whereas at a fiber‐to‐field angle of 0° the T_2_* values were lowest (T_2_* = 18.8 ± 1.3 ms). At 90°, T_2_* values were found to be in between, with mean values of T_2_* = 24.2 ± 1.8 ms.

**Figure 3:**
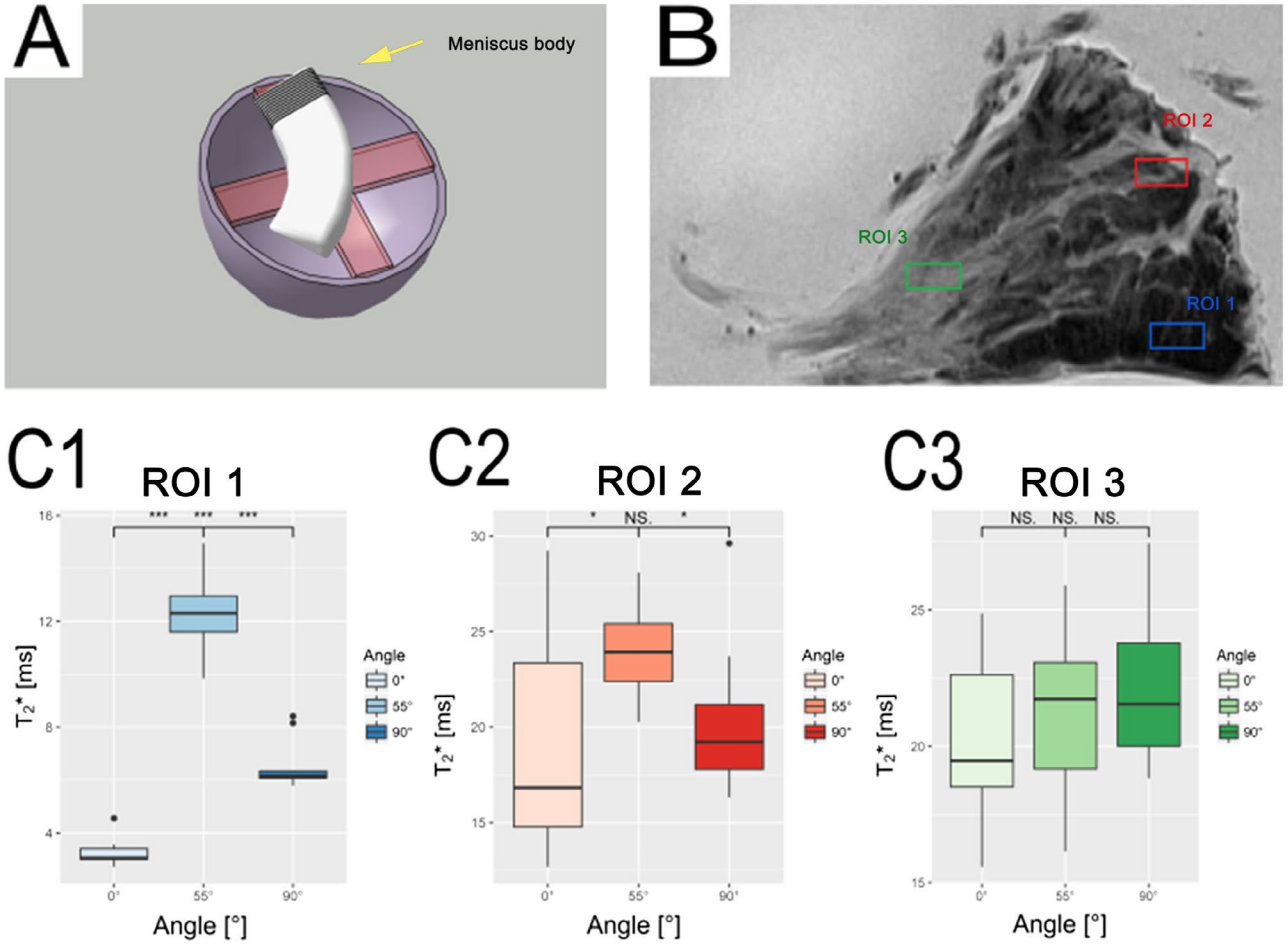
A, Schematic drawing of the position of the 10 representative coronal slices in the body of the meniscus. The meniscus sample was not complete, meaning that the posterior horn was removed during the surgery. B, Representative PD‐weighted image depicting the investigated regions. ROI1 (blue) represents tendon‐like structure, ROI2 (red) represents fibrous‐like tissue from the external circumference, and ROI3 (green) marks fibrous‐like tissue from the internal circumference. C1–C3, Box whisker plots: 10 consecutive slices from the base of the meniscus were analyzed. ROI1: tendon‐like structure; ROI2: fibrous structure from the external circumference; ROI3: fibrous structure from the inner zone. The bold line near the middle of the boxes indicates the median. The bottom of the boxes indicates the 25th percentiles, whereas the top of the boxes delineates the 75th percentiles. The whiskers comprise the data up to the 1.5 interquartile range (IQR). The dots represent outliers. The asterisks in the box plots denote the level of significance (*P* value) between the groups: not significant (NS) = (*P* > 0.05); ^*^(*P* ≤ 0.05); ^**^(*P* ≤ 0.01); ^***^(*P* ≤ 0.001)

There was zonal variation in T_2_* values, which was highly dependent on the orientation of the collagen fibers to the magnetic field. This is depicted by ROIs in a representative proton‐density‐weighted image of the meniscus body (Figure 3B). The results of the voxel‐by‐voxel evaluation are presented in box plots (Figure 3 (C1–C3)).

Tendon‐like structure located in the external circumference (depicted as blue ROI in Figure 3B) showed the strongest orientational dependence, reflecting the highly anisotropic collagen fiber architecture of these structures and the consequential incomplete averaging of dipolar coupling (Figure 2 (B1‐B3), 3 C1). At the magic angle, the mean T_2_* value of this region was 12.19 ± 1.56 ms, whereas at 0° the mean value was 3.3 ± 0.5 ms, which equals an approximate 400% difference between values at these orientations. In contrast, fibrous‐like tissue from the external circumference and fibrous‐like tissue from the internal circumference showed less angle‐dependent changes (Figure 2 (B1‐B3), 3 (C2‐C3)).

**Figure 4:**
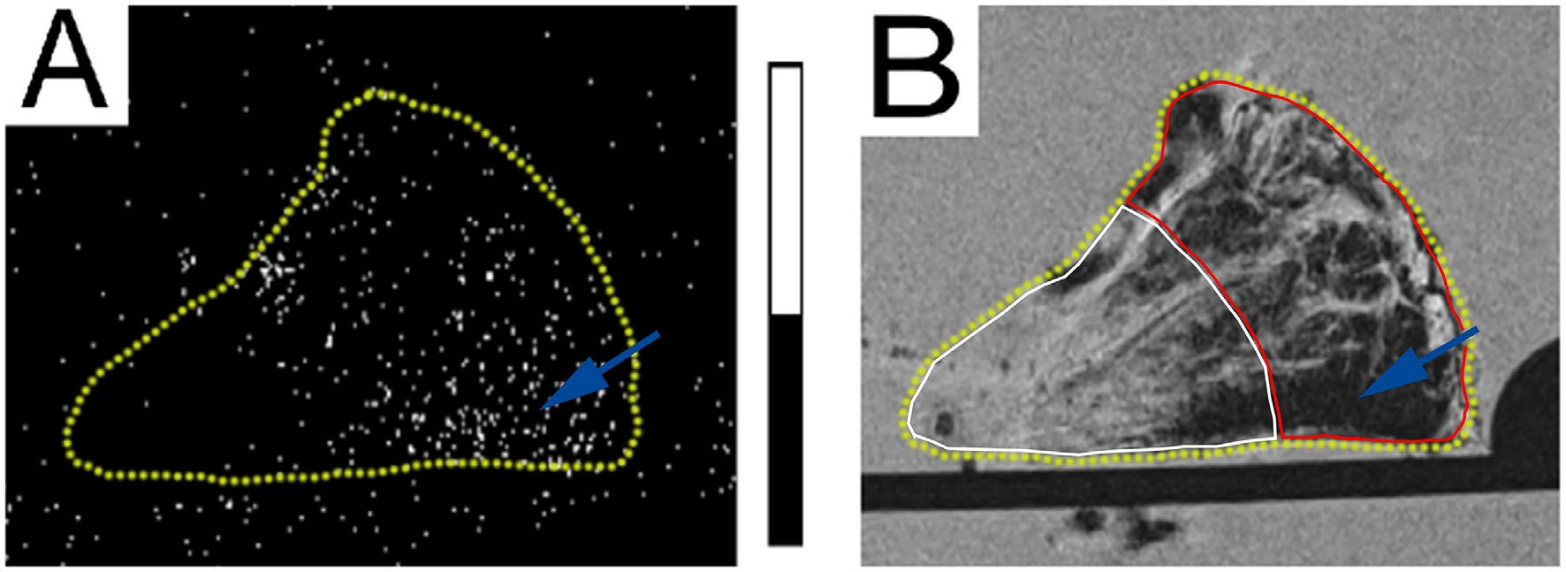
A, Shows a binary map of the pixels with biexponential (white) and monoexponential (black) signal decay when tested with AIC_C_ at a fiber‐to‐field angle of 0°. B, Shows a morphological image with TE of 13.39 ms. Biexponential pixels are primarily found in the tendon‐like region (blue arrows) of the meniscus. The yellow dotted line in both images delimits the meniscus surface from the surrounding water. In case a large ROI‐based analysis is performed (here depicted in B as red and white ROI for red zone and white zone of the meniscus, respectively) similar to what is used in in vivo studies, then the T_2_* decay is preferentially biexponential for all 10 consecutive slices as evaluated by AIC_C _and F‐test

Voxel‐wise analysis, using AIC_C_ and F‐tests, revealed that biexponential pixels can only be found in the tendon‐like region of the meniscus. Exemplarily, Figure 4A shows one representative binary map depicting pixels that can be considered biexponential according to AICc. Figure 4B shows the corresponding morphological echo image with a TE of 13.39 ms.

For ROI‐averaged analysis, the SNR is increased by approximately a factor of 10 to 100, because SNR adds by $\sqrt{n}$,^40^ where n is number of voxels. The results of the ROI‐averaged analysis of experiment 2 are summarized in Table 2. At a fiber‐to‐field orientation of 0°, the tendon‐like region and the fibrous tissue from the external circumference (ROI2) showed preferential biexponential decay as statistically evaluated by AIC_C _and F‐test. In contrast, the fibrous tissue region (ROI3) showed barely any biexponential decay in any orientation (≤10%).

**Table 2:** Summary of results of the orientational ROI averaged analysis of experiment 2

**Region**	**Angle** **[°]**	**T** **_2_** * **_m_** **[ms]**	**T** **_2_** * **_s_** **[ms]**	**T** **_2_** * **_l_** **[ms]**	**F** **_s_** **[%]**	**AIC** **_C_** **[%]**	**F‐test** **[%]**
ROI1	0°	2.08 ± 0.44	1.34 ± 0.49	4.50 ± 1.98	56.14	80	80
						
	55°	9.98 ± 1.92	1.43 ± 0.83	11.80 ± 2.73	14.28	50	40
						
	90°	7.15 ± 0.35	6.59 ± 0.60	29.92 ± 13.88	84.12	0	0
							
ROI2	0°	9.16 ± 1.31	1.31 ± 0.80	10.69 ± 1.30	21.69	60	60
						
	55°	18.19 ± 2.11	0.41 ± 0.53	19.45 ± 2.19	21.30	10	0
						
	90°	15.40 ± 1.65	7.08 ± 6.33	18.85 ± 5.80	23.55	0	0
							
ROI3	0°	20.23 ± 4.48	6.02 ± 7.62	22.14 ± 5.40	18.11	10	10
						
	55°	19.09 ± 5.05	6.95 ± 9.13	20.29 ± 2.92	12.72	0	0
						
	90°	22.83 ± 7.83	11.31 ± 6.72	24.73 ± 4.14	11.15	0	0

ROI1 refers to tendon‐like structure, ROI2 represents fibrous‐like tissue from the external circumference, and ROI3 marks fibrous‐like tissue from the internal circumference. Mean monoexponential T_2_* values (T_2_*m) as well as short and long component values of the biexponential analysis, T_2_*_s_ and T_2_*_l_, respectively, from 10 consecutive slices are provided. The short T_2_ fraction is given as F_s_. The percentage of ROIs from these 10 consecutive slices showing preferential biexponential decay behavior (according to AIC_C_ and F‐test) is given for each of the 3 ROIs and orientations.

For large ROIs of the white zone and red zone, as exemplarily shown in Figure 4, the T_2_* decay showed biexponential decay for all 10 consecutive slices as evaluated by AIC_C _and F‐test.

## 4 DISCUSSION

Mono‐ and biexponential T_2_* mapping was performed on five human meniscus samples using ultrashort echo time and microscopic in‐plane pixel resolution. To this end, for the first time, the relationship between T_2_* values and collagen alignment of one human meniscus sample was studied based on a T_2_* analysis at a series of circumferential fiber to magnetic field orientations. All MR findings were compared to histological findings.

Our study showed a zonal analysis of spatially matched T_2_* maps and histology images of the human meniscus with a resolution that, to our knowledge, has not been reported yet. Moreover, our study shows a strong orientational anisotropy of T_2_* values; especially for the highly ordered tendon‐like region of the meniscus. Furthermore, T_2_* decay showed no clear biexponential decay pattern, in contrast to the findings of previous studies (e.g., Diaz et al.^10^).

This study shows that ultrahigh field strength (7 T), in combination with a small‐sample MR microimaging system, allows to visualize the human meniscus and its ultrastructure with microscopic in‐plane pixel resolution and high SNR in relatively short scan time (i.e., 10 minutes for one single‐echo measurement). This is both attributed to the high field strength (B_0_), offering an SNR increase proportional to B_0_^7/4 ^in theory^41^ in combination with small‐volume coils (19 and 39 mm), where SNR is approximately inversely proportional to the coil diameter (SNR ~ 1/d). As a result, voxel sizes of 60 × 60 × 400 μm^3^ (experiment 1) and 67 × 67 × 400 μm^3^ (experiment 2) became feasible, yielding a microscopic pixel resolution along two spatial directions. Overall, this yields an increase in the sensitivity by one to two orders of magnitude, as compared to conventional whole‐body MRI with large coils.

Nevertheless, up until now, MR microscopy of short T_2_/T_2_* musculoskeletal tissue such as the meniscus is still rarely performed because of lack of hardware availability close to clinical applications (scanner, microimaging system, and coils) and sequence limitations. However, it is well known that these very‐high‐resolution measurements of highly organized collagen tissues are important in the field of MRI, because the results can help explain different intrinsic MR properties (T_2_, T_2_*, and T_1ρ_) and appearances of tissue in MRI (e.g., with change in fiber‐to‐field orientation).

Bae et al.^42^ previously showed high nominal spatial resolution (voxel size = 130 × 130 × 130 µm^3^) human meniscus images using a 2D and 3D ultrashort echo time (UTE) pulse sequence. They found that using UTE sequences and shortest, sub‐ms TE allows to detect high signal from all the meniscus substructures and that the fibrillar network is better depicted at TE ~ 5 ms, which is in accord with the findings presented in our study.

In our study, a 3D variable echo time (3D vTE) sequence was used to acquire images with ultra short echo time, which allowed to gain signal from highly ordered collagen tissue such as the meniscus. Moreover, this sequence uses a rectilinear k‐space sampling scheme, where it differs from UTE sequences, which mostly use center‐out radial trajectories. Consequently, the 3D vTE sequence is less prone to artefacts from k‐space trajectory errors.^22^ Moreover, it allows faster measurement compared to radial acquisition, which have an inherently less‐efficient acquisition scheme.^43^

The sequence also benefits considerably from the strong gradients (750 mT/m) of the 7 T microimaging system and allows even shorter echo time (~0.4 ms) compared to 7 T in vivo whole‐body imaging, where the minimum effective echo time is around 0.8 ms.^7^

T_2_* maps and PLM images of human meniscus samples show noticeable similarities, indicating that T_2_* maps are very sensitive to the heterogeneous ultrastructure in terms of collagen fiber density and their orientation. With an in‐plane pixel resolution of 60 × 60 μm^2^, we were able to visualize and quantify (in terms of T_2_* values): fibrous bundles, circumferential fibers, and the laminar layer.

Up until now, T_2_* mapping of the meniscus using microscopic resolution, in combination with ultrashort echo time and comparison to histology, has not been performed yet. However, a comparison between T_2_* values of comparatively lower resolution data (voxel size = 270 × 270 × 2000 μm^3^) and a histological score was performed by Williams et al.^6^ In accord with their study, we found that regions of advanced degeneration in the meniscus, such as fibrous remodeling and extensive fraying, clearly show higher T_2_* values.

Furthermore, Williams et al. claimed that UTE is, strictly speaking, not required to study meniscus T_2_* relaxation. Nevertheless, it was noted that the use of sub‐ms echo times improves both capture and curve fitting of the short T_2_* component (i.e., <6 ms) and therefore providing increased sensitivity to subtle differences between meniscus regions that may help to detect earlier changes to meniscus health.

In our study, we found that thick fibrous bundles from the joint capsule and zones of extensive fraying show higher T_2_* values. Evidently, for these structures UTE is not necessary. However, for highly ordered circumferential fibers, which tend to have short T_2_* values around 5 ms (depending on the fiber‐to‐field angle), UTE likely leads to more reliable results when studying T_2_* relaxation, because considerable signal decay already occurs within the first millisecond.

In experiments 1 and 2, the number of pixels that can be considered to feature biexponential decay was extremely low as evaluated with AIC_C_ and F‐test. This seems to be in contradiction to the results of high‐resolution T_2_* mapping in vivo.^7^ However, we assume that this can possibly be attributed to the compartmentalization of the meniscus. With increasing voxel size, the probability of voxels covering multiple tissue types of the meniscus (e.g., tendon‐like collagen fibers and fibrous tissue together) will be increased.

However, it should also be noted that in earlier biexponential/bicomponent studies (e.g., Diaz et al.^10^ and Juras et al.^7^) no model testing between mono‐ and biexponential model was performed. Therefore, it cannot be ruled out, that their biexponential interpretations suffer from misinterpretation attributable to overfitting.

The number of echo times used in experiment 1 (9 TEs) was similar to what was used in previous bicomponent studies^7^, ^44^; however, this number can still be considered low for biexponential analysis. Therefore, the low number of biexponential pixels in experiment 1 could also represent a consequence of these experimental constraints. In experiment 2, we accounted for these experimental restrictions by increasing the number and interval of echo times to cover longer T_2_* relaxation times. Nevertheless, in experiment 2, biexponential T_2_* decay behavior was still scarce on a voxel‐by‐voxel basis. On a small ROI‐averaged basis, which incorporates higher SNR values by approximately a factor of 10, the tendon‐like region showed preferentially biexponential signal decay for a fiber‐to‐field angle of 0°, but not for an angle of 55°. At the magic angle (55°), we found a balance between mono‐ and biexponential decay. We hypothesize here, based on the literature,^12^ that the short component of T_2_* (bound water) is subject to a stronger orientation dependence than the long component attributable to unaveraged dipolar coupling. This hypothesis could explain, to some extent, the vanishing of the biexponential decay at the magic angle: If it is assumed that the short component (only) exhibits an increase of T_2_* toward the long T_2_* of the other tissue component approaching the magic angle orientation, then the two components cannot be adequately differentiated anymore by the fitting algorithm. However, our results on ROI‐averaged basis indicate that the T_2_* decay at magic angle still showed more biexponential decay behavior than at 90°, which is not supported by this hypothesis.

For very large ROIs covering the red zone and white zone of the meniscus, we found a preferential biexponential decay when measured at a fiber‐to‐field angle of 0°, which indicates that bicomponent analysis could be more relevant for cases where a combination of long and short T_2_/T_2_* tissues are analyzed together in an ROI (i.e., low‐resolution clinical/translational images) and less important when each structure can be resolved using microscopic resolution imaging.

We showed that monoexponential T_2_* values varied up to 400% to 500% (for voxel‐wise– and ROI–averaged analysis, respectively) with orientation to the magnetic field, especially in the highly anisotropic tendon‐like parts of the meniscus. This is in line with the results of Henkelmann et al.,^12^ who measured 6 different tissues, including bovine Achilles tendon, and found that particularly short relaxing components of highly organized tissues are affected by relaxation anisotropy attributable to incomplete averaging of dipolar coupling.

Moreover, the results are in accord with the results of Krasnosselskaia et al.,^14^ who measured bovine digital flexor tendon and found a factor of 6 change in signal intensity with change in fiber‐to‐field orientation from the “magic angle” of 54.7° to 0°.

In our study, we found similar orientational fiber‐to‐field T_2_* behavior, as expected on the basis of previous magic‐angle studies. Highest T_2_* values were found at 55°, the magic angle, where most of the water‐related protons precess at comparably similar frequencies. In contrast, lowest values were found at 0°, where the dipolar interaction is at a maximum. Mobile and immobile protons tend to precess at different frequencies, which results in a decrease of effective T_2_ and T_2_* values. At 90°, the combined effect of the two magic angles, 55° and 125°, does not allow to reach a similar decrease of 0°, and therefore the T_2_* values found for this angle are at a level somewhere between the 0° and 55° effect.^17^

In our opinion, this fiber‐to‐field angle dependence has strong clinical implications not only for quantitative T_2_/T_2_* mapping, but also for meniscus measurements in general. The reason for that is that T_2_* values, T_2_ values, and also signal‐intensity values of morphological sequences (be it T_2_–, T_1_–, or PD–weighted images), always (naturally) have some contribution from transversal relaxation time and are affected because of knee positioning in the coil (and thus the fiber‐to‐field angle). Here, we performed this orientational analysis of the bulk meniscus fibers in terms of T_2_* values for the first time, and we showed that for these highly ordered fiber bundles, the angle dependence can be highly significant. Moreover, we believe this should be appropriately taken into consideration in every meniscus MRI measurement to standardize clinical imaging protocols by ensuring similar knee angle and thus fiber‐to‐field angle and to subsequently avoid misinterpretation.

The findings in this study can also be applied to lower field strength, such as 1.5 and 3 T, where likely a similar bicomponent behavior would be observed. However, lower SNR in lower field strengths will inevitably limit the precision of the bicomponent analysis.

MRI has become the most important tool to assess meniscus health and degeneration and is used for preoperative assessment as well as postoperative follow‐up. So far, in clinical routine this assessment is restricted to morphological MRI. However, newly developed quantitative techniques, such as T_2_* mapping, might not only improve diagnosis of early degeneration preceding a subsequent tear, but also allow for exact monitoring of disease and repair, which would, in turn, allow for quantitative studies on different surgical treatment options. Both would lead to significant benefits for patients.

There are some limitations in this study. First, within the scope of this MR microscopy study, only a small sample size has been investigated for looking into the main effects of orientation dependence and differentiation between bi‐ and monoexponential T_2_* decay. Consequently, a correlation between T_2_* values and a histological score has not been studied. A study including healthy human menisci may address this in future studies; however, healthy ex vivo human meniscus samples were not available for this study. Second, the SNR (~35) and the previously mentioned number of echo times were relatively low for a thorough biexponential analysis, which potentially affected the outcome of the voxel‐wise biexponential analysis. Hence, optimal echo time distribution, number of echo times, and optimal and minimum SNR values for a precise biexponential T_2_* mapping of the meniscus should be evaluated in a future biexponential simulation study.

## 5 CONCLUSION

In our study, we demonstrated the highest‐resolution meniscus measurements to date allowing the identification of meniscus substructures and structural alterations and to quantify T_2_*. We found no clear biexponential decay behavior for the meniscal substructures on a small‐sized voxel basis. Moreover, a strong orientational dependence of T_2_* decay reflecting the anisotropic properties of the meniscal collagen fibers was demonstrated. MR microscopy can help clarify the complexity of the meniscus substructures and might help in the interpretation of moderate resolution T_2_/T_2_^*^‐weighted images and T_2_* values. The results of this study may support future studies using T_2_* mapping techniques to identify patients suffering from early degeneration and monitor therapeutical interventions.
